# The efficacy and dosimetry analysis of CT-guided ^125^I seed implantation assisted with 3D-printing non-co-planar template in locally recurrent rectal cancer 

**DOI:** 10.1186/s13014-020-01607-2

**Published:** 2020-07-25

**Authors:** Lu Wang, Hao Wang, Yuliang Jiang, Zhe Ji, Fuxin Guo, Ping Jiang, Xuemin Li, Yi Chen, Haitao Sun, Jinghong Fan, Weiyan Li, Xu Li, Junjie Wang

**Affiliations:** Department of Radiation Oncology, Peking University Third Hospital, 49 North Garden Road, Haidian District, Beijing, 100191 People’s Republic of China

**Keywords:** Locally recurrent rectal cancer, 3D-printing non-co-planar template, Efficacy, ^125^I seed implantation, Dosimetry

## Abstract

**Background:**

Locally recurrent rectal cancer (LRRC) after surgery or external beam radiotherapy (EBRT) is a serious challenge for which no standard treatment is defined. In the present study, we investigated the feasibility of computed tomography (CT)-guided radioactive ^125^I seed (RIS) implantation assisted with three-dimensional printing non-coplanar template (3D-PNCT) in LRRC patients who previously received surgery or EBRT.

**Methods:**

Sixty-six patients with LRRC treated by CT-guided RIS implantation in our institute from December 2015 to May 2019 were included. The treatment procedure included: preoperative CT localization, planning design, the printing of 3D individualized template, CT-guided RIS implantation assisted with 3D-PNCT, and postoperative dose evaluation. Therapeutic outcomes including local control (LC) and overall survival (OS) were retrospectively evaluated, as well as side effects.

**Results:**

All the patients had previously received surgery or EBRT. The median follow-up time was 12.2 (range, 2.5–35.9) months. The median radioactive activity of a single RIS was 0.6 (range, 0.43–0.72) mCi. The median number of RIS was 60, ranging from 10 to 175. The dosimetric parameters included D90 (140.7 ± 33.1) Gy, D100 (90.3 ± 138.6) Gy, and V100 (91.0 ± 13.3) %. Pain relief was achieved in 85.1% (40/47) of patients. Besides, 9.1% (6/66) of patients had severe side effects (≥grade 3), including perianal skin ulcer in 1 case, fistula, radiation proctitis, and intestinal obstruction each in two cases. Median OS time was 14.7 (95% confidence interval (CI): 13.0–16.3) months, and median LC time was 12.2 (95% CI: 9.1–15.2) months. Univariate analysis revealed that when D90 > 130 Gy or D100 > 55 Gy or V100 > 90%, the LC time was remarkably prolonged. However, none of the parameters significantly affected OS.

**Conclusions:**

CT-guided RIS implantation assisted with 3D-PNCT is an effective and safe salvage treatment strategy for patients with LRRC after EBRT or surgery. D90, D100, and V100 can be used as prognostic predictors.

**Trial registration:**

NCT03890926.

## Background

Locally recurrent rectal cancer (LRRC) is defined as an intrapelvic recurrence following a primary rectal cancer resection, with or without distal metastasis. Concurrent preoperative chemoradiotherapy followed by total mesorectal excision (TME) surgery plus systemic chemotherapy is the first recommended standard treatment for patients with resectable rectal cancer (stage II-III) [1–3]. This treatment strategy has significantly decreased local recurrence rate and increased disease free survival (DFS) rate. However, it was reported that 2.4–10% of patients experienced local relapse [4–6]. Patients may suffer from local compression symptoms, severely influencing their daily life.

Several treatment modalities have been reported for LRRC after external beam radiotherapy (EBRT) or surgery, while their outcomes have still remained unsatisfactory. Radical (R0) resection was proved to be effective, while the majority of patients were not ineligible candidates. Computed tomography (CT)-guided ^125^I seed implantation has been found as an effective and safe treatment option for recurrent rectal cancer after surgery or EBRT accompanied by mild adverse reactions [7]. The 2016 National Comprehensive Cancer Network (NCCN) Clinical Practice Guidelines in Oncology has recommended radioactive ^125^I seed (RIS) implantation for the treatment of LRRC [8]. However, the efficiency and accuracy of traditional ^125^I seed implantation under the guidance of imaging mainly relied on operators’ experience. Misplacement of RIS would lead to unsatisfactory outcomes. Thus, we, in the present study, concentrated on the application of CT-guided RIS implantation assisted with three-dimensional printing non-co-planar template (3D-PNCT) for LRRC patients who previously received surgery or EBRT. The therapeutic efficacy was evaluated, and dosimetric parameters related to prognosis were analyzed.

## Patients and methods

### Data collection

From December 2015 to May 2019, data of 66 patients with LRRC who were treated with 3D-PNCT-assisted CT-guided RIS implantation, and previously received surgery or EBRT were collected. The study was approved by the Ethics Committee of our hospital (Approval No. IRB00006761). The inclusion criteria were as follows: (1) patients who were histopathologically diagnosed as LRRC; (2) history of EBRT or surgery, incapable of re-irradiation or resection; (3) tumor diameter ≤ 7 cm; (4) suitable puncture path; (5) tolerant to puncture and anesthesia; (6) no bleeding tendency; (7) Karnofsky performance status > 70; (8) life expectancy ≥ 3 months.

### Treatment procedures

#### Preoperative CT localization

All patients were fixed with a vacuum pad and underwent spiral CT (Brilliance BigBore, Philips, Amsterdam, the Netherlands) 2 days before surgery to locate the tumor. The optimal position (prone or supine position) was chosen according to the tumor site to facilitate accurate needle insertion. Then, the positioning lines were marked on the surface of skin.

#### Preoperative planning design

**Fig. 1:**
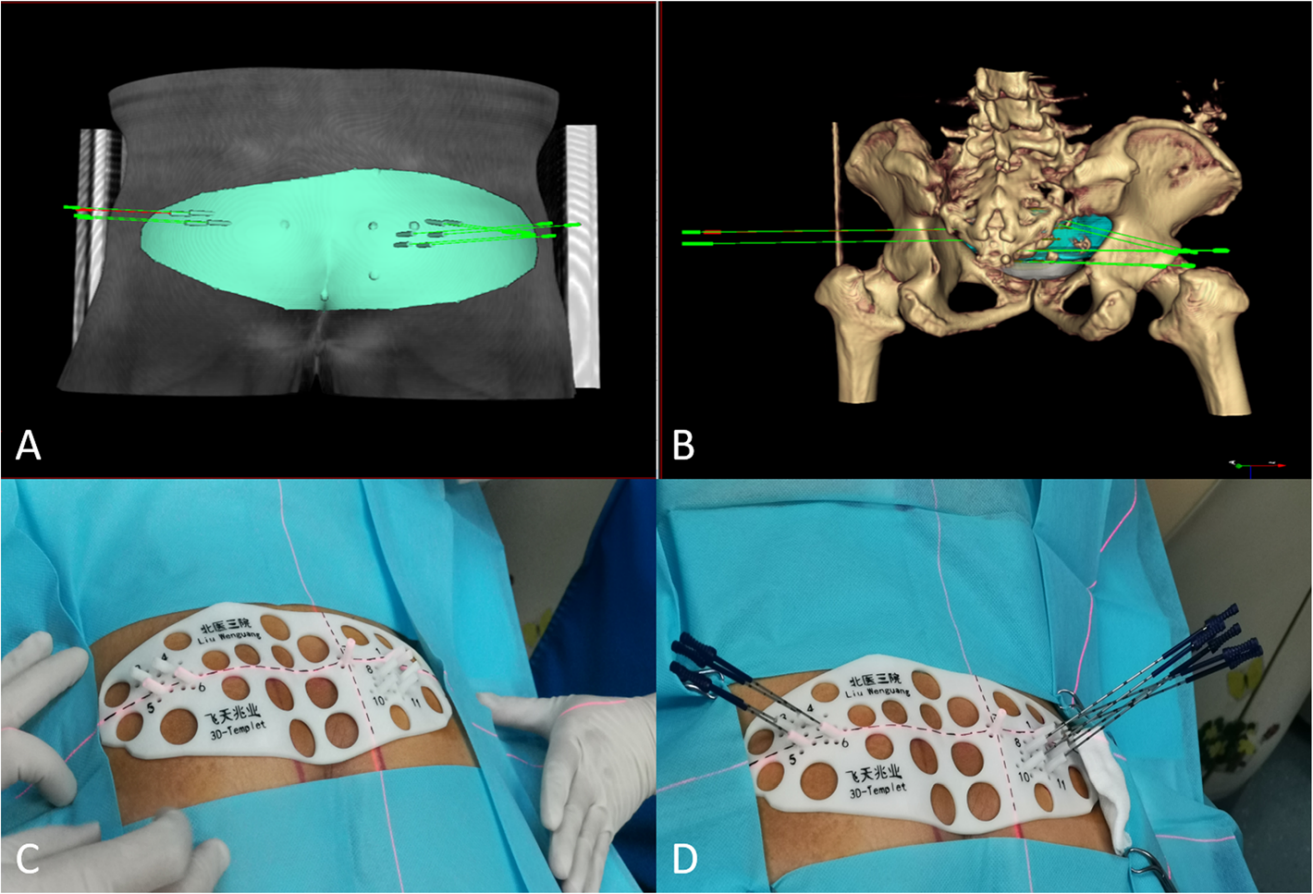
(**a**) (**b**) Preoperative simulation of seeds implantation assisted with 3D-PNCT in B-TPS. **c** Fixation of 3D-PNCT on the surface of the skin according to the positioning markers. **d** The needles were inserted to the planned depth through the channels in 3D-PNCT. 3D-PNCT: 3D-printing non-co-planar template; B-TPS: brachytherapy treatment planning system

CT data were transmitted to the Brachytherapy Treatment Planning System (B-TPS), which was designed by Beijing Astro Technology Co., Ltd. and the Beijing University of Aeronautics and Astronautics. The preoperative planning design included the following steps: delineation of the gross tumor volume (GTV) and organs at risk (OARs); determination of prescription dose (110–160 Gy) and RIS (6711_1985; Shanghai GMS Pharmaceutical Co., Ltd.) activity; design of needle channels; simulation of seeds distribution; and calculation of dosimetric parameters (Fig. 1a and b).

#### Printing of 3D individualized template

According to the treatment planning system design, an individualized 3D-PNCT was printed by a 3D rapid-prototyping equipment with photo-curable resins. The template contained the superficial anatomic characteristics of the treatment area, positioning markers and needle channels.

#### CT-guided RIS implantation assisted with 3D-PNCT

Local or spinal anesthesia was induced. The 3D-PNCT was fixed on the surface of the skin according to the positioning markers (Fig. 1c). Then, seed needles were inserted into the channels of the 3D-PNCT (Fig. 1d). Before the needles were inserted into the body, an enhanced CT scan was performed to visualize directions of the needles. The purpose was to confirm whether the directions of the needles in the template were consistent with the preoperative plan, and whether there were blood vessels or organs in the direction of the needles. After CT verification, all needles were inserted into the body at the planned depth. Further CT scan was carried out to visualize whether positions of the needles were consistent with the preoperative plan. After the CT verification, ^125^I seeds were implanted into the tumor using a Mick gun (Mick Radio-Nuclear Inc., Mount Vernon, NY, USA) according to the preoperative plan.

#### Postoperative dose evaluation

**Fig. 2:**
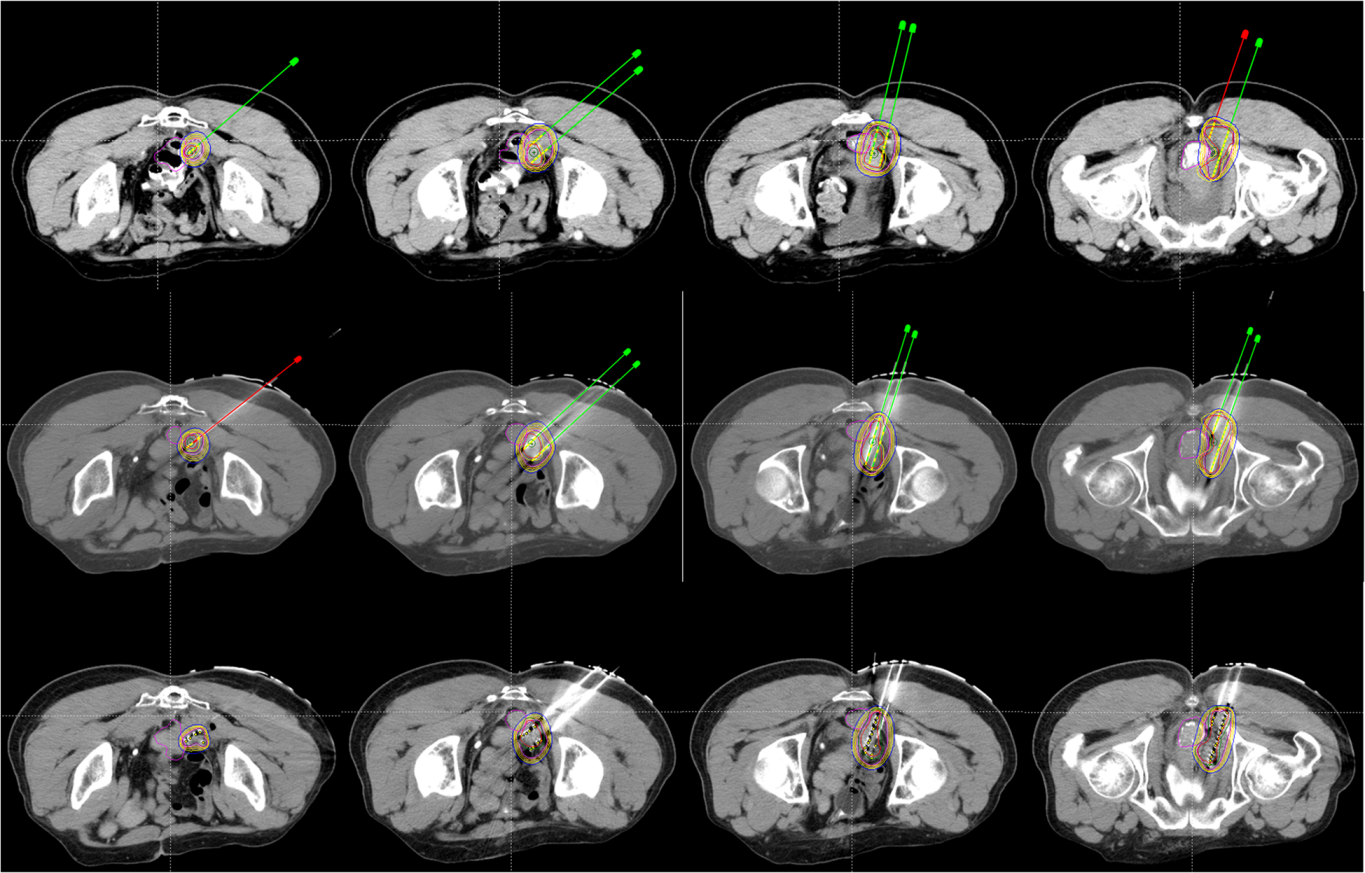
The first line presents the preoperative plan. The second line presents the actual positions of the needles before seeds implantation during the operation. The third line presents the actual distribution of seeds after implantation

A CT scan was performed immediately after implantation of ^125^I seeds to verify the distribution of RIS and evaluate the actual dose of the targets (Fig. 2). The dosimetric parameters included: 1) dose delivered to 90% of GTV (D90), 2) dose delivered to 100% of GTV (D100), 3) percentage of GTV receiving 100% of the prescription dose (V100), 4) percentage of GTV receiving 150% of the prescription dose (V150), 5) percentage of GTV receiving 200% of the prescription dose (V200), 6) the conformal index (CI), 7) external index (EI), and 8) homogeneity index (HI).

### Follow-up

The patients were followed-up every 3 months since the time of implantation. The disease condition was evaluated by routine blood test, blood biochemical test, tumor markers test, abdominal CT, chest CT, and pelvic magnetic resonance imaging (MRI). Tumor response was assessed by the RECIST guideline (version 1.1) [9]. Complete response (CR) indicated that all the known tumor lesions disappeared. Partial response (PR) was defined as at least a 30% decrease in the sum of diameters of tumor lesions. Progressive disease (PD) was defined as an increase of 20% or more in the sum of diameters of tumor lesions. Stable disease (SD) was defined as a less than 30% decrease or a less than 20% increase in the sum of diameters of tumor lesions. Local control was defined as no PD in the implanted tumor site. The pain was graded by the Numeric Rating Scale (NRS) [10]. The side effects were evaluated by the toxicity criteria of the Radiation Therapy Oncology Group (RTOG) [11].

### Statistical analysis

All the statistical analyses were carried out with SPSS 18.0 software (IBM, Armonk, NY, USA). Kaplan–Meier survival analysis was used to estimate local control (LC) time and overall survival (OS) time. The receiver operating characteristic (ROC) curve was used to analyze the optimal values of dosimetric parameters and the patients were divided into two groups. Log-rank test was employed to compare the differences between the two groups. Cox proportional hazards regression model was used to analyze confounding factors that affected LC and OS. *P* value < 0.05 was considered statistically significant. The curves were plotted by GraphPad Prism 5.0 software (GraphPad Software Inc., San Diego, CA, USA).

## Results

### Patients’ general information

The median follow-up time was 12.2 (2.5–35.9) months. The patients’ median age was 56 (range, 32–79) years old. All the patients received EBRT or surgery before RIS implantation. Herein, 63 patients underwent surgery and 3 patients had no history of surgery. Besides, 62 of 66 patients had received EBRT previously. All the patients were treated with chemotherapy previously. The patterns of recurrent tumors included 31 (47.0%) cases with sacral invasive, 19 (28.8%) cases with lateral invasive, and 16 (24.2%) cases with localized type. The patients’ baseline characteristics are listed in Table 1.

**Table 1:** Patients’ characteristics at baseline

Characteristic	Value
Number	66
Sex, n (%)	
Male	38 (57.6%)
Female	28 (42.4%)
Age (in years), median (range)	56 (32–79)
≤ 40	3 (4.5%)
40–50	17 (25.8%)
50–60	26 (39.4%)
>60	20 (30.3%)
Karnofsky performance score (%), median (range)	81 (60–90)
Recurrent pattern ^a^, n (%)	
Sacral invasive type	31 (47.0%)
Lateral invasive type	19 (28.8%)
Localized type	16 (24.2%)
Histopathology	
Adenocarcinoma	63
Adenosquamous carcinoma	3
Previous surgery	
None	3
Once	49
Twice	14
Total dose of previous EBRT (Gy)	
< 50	7
50–100	47
> 100	12
Courses of previous EBRT	
0	4
1	46
2	13
3	3
Gross tumor volume (ml), average ± standard deviation	46.0 ± 48.9
Time from tumor recurrence to RIS implantation (in months), median (range)	16 (0.5–62)

^a^: Sacral invasive type: invasion of lower sacrum (S3, S4, S5), coccyx or periosteum; Lateral invasive type: invasion of upper sacrum (S1, S2), sciatic nerve, greater sciatic foramen or lateral pelvic wall; Localized type: invasion of surrounding pelvic organs or tissue s[12]. EBRT: external beam radiotherapy; RIS: radioactive ^125^I seed

The median radioactive activity of a single RIS was 0.6 (range, 0.43–0.72) mCi. The median number of RIS was 60, ranging from 10 to 175. Postoperative dosimetric parameters included: D90 (140.7 ± 33.1) Gy, D100 (90.3 ± 138.6) Gy, V100 (91.0 ± 13.3) %, V150 (68.9 ± 16.7) %, V200 (45.1 ± 17.0) %, HI (0.25 ± 0.14), EI (3.2 ± 6.9), and CI (0.49 ± 0.22).

### LC time and OS time

**Fig. 3:**
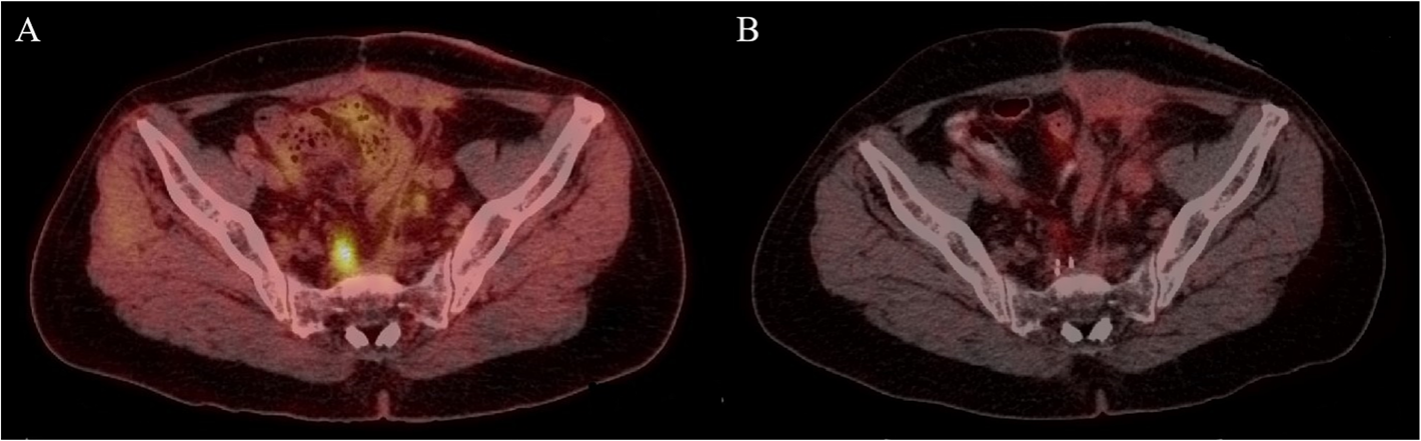
Stage III, T3N2M0, adenocarcinoma, presacral recurrence, previous Dixon’s operation, EBRT: DT 50.4 Gy before. PET-CT images (A) before seeds implantation, (B) 3 months after seeds implantation. EBRT: external beam radiotherapy; PET-CT: positron emission tomography-computed tomography

Median LC time was 12.2 (95% confidence interval (CI): 9.1–15.2) months. The LC rate was 62.3% for 1 year and 37.3% for 2 years. The rates of CR, PR, SD, and PD were 18.2% (12/66), 66.7% (44/66), 10.6% (7/66), and 4.5% (3/66), respectively, 3 months after the seed implantation (Fig. 3). The objective remission rate (ORR) was 84.9%.

Additionally, 24 (36.4%) patients were still alive and 42 (63.6%) patients died at the end of the follow-up. Besides, 31.0% (13/42) patients died of local recurrence, 66.7% (28/42) patients died of metastasis, and 2.3% (1/42) patients died of non-tumor causes. The median OS time was 14.7 (95% CI: 13.0–16.3) months. The one-year and two-year OS rates were 60.5 and 25.5%, respectively.

### Symptom relief and side effects

The pain relief rate was 85.1% (40/47). The remission rate was 50% (3/6) for dysuria and 50% (1/2) for frequent micturition. For implantation-related complications, the implantations were successful in all cases. No serious complications occurred during the perioperative period. One patient had fever on the day after the implantation, while the temperature returned to baseline with the use of antibiotics. It was unveiled that the fever was caused by a pelvic infection. Another patient had an episode of pain in the area of implantation right after the implantation, whereas the pain was relived soon without intervention. The volume of blood loss during surgery was low in all the patients. No patient showed RIS immigration during the follow-up. Concerning long-term side effects, 7.6% (5/66) of patients had side effects (≤ grade 2), including 1 (1.5%) case with perianal pain, 1 (1.5%) case with diarrhea, and 3 (4.5%) cases with neuropathy. Furthermore, 9.1% (6/66) of patients had side effects (≥ grade 3), including 1 (1.5%) case with perianal skin ulcer, 2 (3.0%) cases with fistula, 2 (3.0%) cases with radiation proctitis, and 2 (3.0%) cases with intestinal obstruction.

### Statistical analysis

**Fig. 4:**
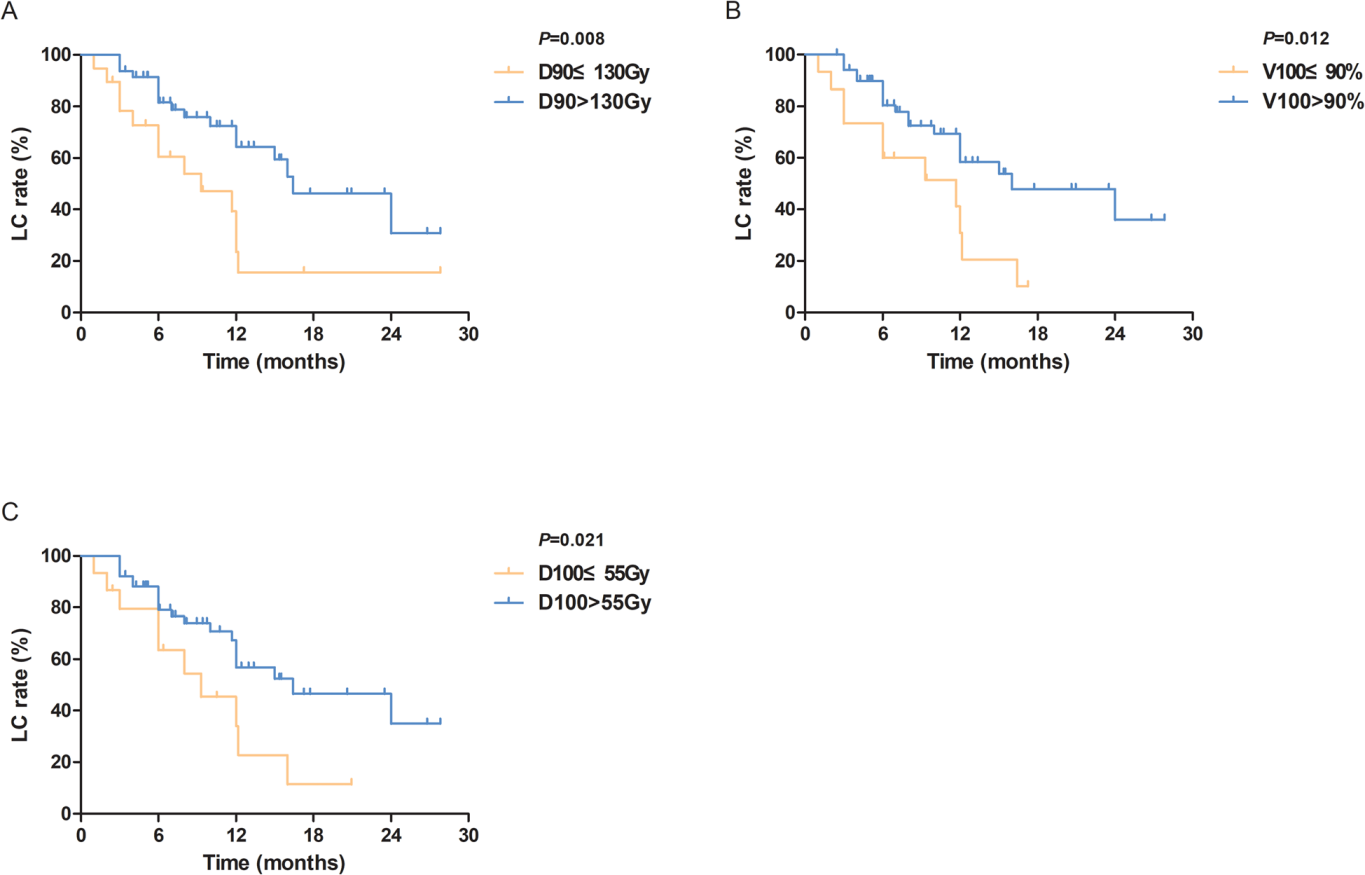
Kaplan–Meier plots of (A) LC curve of different D90; (B) LC curve of different V150; (C) LC curve of different D100. LC: local control; D90: dose delivered to 90% of GTV; D100: dose delivered to 100% of GTV; V100: percentage of GTV receiving 100% of the prescription dose

Regarding LC time, the univariate regression analysis suggested that D90, D100, and V100 significantly influenced LC time (*p* = 0.004, 0.018, and 0.023, respectively) (Table 2). All the above-mentioned factors and other factors (*p* < 0.2) were enrolled in the multivariate regression analysis of LC time, and no statistical difference was found. D90, D100, and V100 were divided into two groups according to the ROC curves, respectively. The univariate regression analysis showed that when D90 > 130 Gy or D100 > 55 Gy or V100 > 90%, the LC time was remarkably prolonged (*p* = 0.008, 0.021, and 0.012, respectively) (Fig. 4). The one-year LC rates for D90 ≤ 130 Gy, D90 > 130 Gy, D100 ≤ 55 Gy, D100 > 55 Gy, V100 ≤ 90%, and V100 > 90% were 39.3, 72.4, 45.4, 67.4, 41.1, and 69.3%, respectively. However, the univariate regression analysis showed no correlation between OS time and dosimetric parameters.

**Table 2:** Cox univariate analysis of factors influencing LC

Factors	Hazard ratio	95% CI	*p*
D90	0.986	0.977–0.996	0.004
D100	0.981	0.965–0.997	0.018
V100	0.980	0.963–0.997	0.023
V150	0.987	0.968–1.007	0.194
V200	1.002	0.981–1.023	0.860
HI	2.653	0.253–27.832	0.416
CI	0.895	0.202–6.227	0.895
EI	0.990	0.935–1.048	0.723
Activity of a single RIS	0.404	0.002–65.400	0.727
Number of RIS	1.002	0.991–1.013	0.764
Age	0.997	0.964–1.031	0.872
Gross target volume	1.000	0.991–1.009	0.966
Pathological differentiation	0.680	0.190–2.430	0.553
Courses of previous EBRT	0.745	0.380–1.460	0.391
Total dose of previous EBRT	0.997	0.984–1.010	0.621
Time from tumor recurrence to RIS implantation	0.979	0.953–1.006	0.124
Recurrent pattern	1.076	0.699–1.659	0.739

*LC* local control; *D90* dose delivered to 90% of GTV; *D100* dose delivered to 100% of GTV; *V100* percentage of GTV receiving 100% of the prescription dose; *V150* percentage of GTV receiving 150% of the prescription dose; *V200* percentage of GTV receiving 200% of the prescription dose; *HI* homogeneity index; *CI* the conformal index; *EI* external index; *RIS* radioactive ^125^I seed; *EBRT* external beam radiotherapy

## Discussion

As reported previously, in comparison with microscopically irradical resection and macroscopically irradical resection, R0 resection can significantly improve OS, DFS, and LC [13]. Further, neoadjuvant chemotherapy or EBRT can increase the possibility of R0 resection. However, the majority of patients with LRRC are inappropriate for secondary operation due to the involvement of organs or nerves [2]. Furthermore, the second operation is very difficult to perform. After the first operation, the anatomical structure of the pelvis changes and the pelvic cavity adherence is severe, making it challenging for surgeons to distinguish scar tissues from the tumor. A small surgical space remains that makes it more likely to damage organs, cause presacral hemorrhage, and even result in tumor metastasis [2, 14–16].

Although EBRT alone or chemotherapy can relieve symptoms, the 5-year survival rate is still lower than 5% and adverse effects are remarkable. Guren et al. reported 375 patients with LRRC who received reirradiation (hyperfractionated or once-daily chemoradiotherapy) [17]. The median previous irradiation dose was 50.4 Gy. The median reirradiation dose was 30–40 Gy. For patients who received resection after reirradiation, the median OS time was 39–60 months, and the median OS time was only 12–16 months for patients who received palliative reirradiation. The rate of acute toxicity was 9–20%. Lee et al. reported that the rates of acute and late toxicity for reirradiation were 11.7 and 25.5%, respectively [18]. The previous irradiation dose to the normal tissue may has already reached the tolerated dose, which made it difficult for the patients to undergo EBRT again [19]. Besides, previously irradiated tumors were found less sensitive to chemotherapy than tumors that were never irradiated [20]. Recurrent tumor after surgery or EBRT is typically associated with a poor blood supply, leading to poor response to reirradiation and chemotherapy.

Intraoperative radiotherapy (IORT) can radiate the tumor directly while sparing normal tissues. Haddock et al. reported that patients with previous EBRT can be treated with IORT. However, several researchers demonstrated that the LC rate and OS rate of IORT alone were noticeably lower than those of IORT combined with EBRT [21, 22]. In Mayo Clinic, patients, who previously received 30 Gy EBRT, were treated with IORT combined with EBRT. Five-year OS rate was 32%, which was remarkably higher than that of patients who were treated with IORT alone (22%). Furthermore, IORT significantly increased complications related to wound healing, such as wound infection and pelvic abscess. Neuropathy was also a remarkable side effect, which was closely related to the dose of IORT [19, 23]. Besides, whether a patient can receive IORT depends on whether he/she is eligible for surgery.

RIS implantation refers to the implantation of RIS into the tumors during the operation or with the help of imaging equipment. Because of the special physical characteristics of RIS, the dose is inversely proportional to the square of the distance. The dose of the normal tissue around the tumor is dramatically reduced. RIS persistently radiates a low dose of rays (initial dose rate 0.07–0.09Gy/h, t_1/2_ = 60 days) that effectively kill tumor cells without causing a remarkable damage to surrounding normal tissues. Besides, the implantation does not require high physical strength of patients and has no limitation on the dose of previous EBRT, thus, the majority of patients exhibited an acceptable tolerance [7, 24].

Numerous clinical studies pointed out that RIS implantation is a safe and effective approach with few adverse effects. Goes et al. first reported 30 patients with LRRC who were treated with radioactive seeds implantation [25]. The LC rate was 66% for microscopic residual disease and 37.5% for gross residual disease. At the time of the last follow-up, 18 (64%) patients had locally recurrent tumors under control. Martinez et al. studied 29 patients with recurrent colorectal cancer who were treated intraoperatively with ^125^I seed implantation [26]. They received a median minimal peripheral dose (MPD) of 140 Gy. The median time to local failure was 11 months (95% CI 10–12 months) and the median OS time was 18 months (95% CI: 14–22 months). Moreover, 13 (45%) patients experienced 15 toxic events, including 3 (10%) patients with enteric fistula. Wang et al. assessed 13 patients with LRRC who received percutaneous ^125^I seeds implantation with a median MPD of 140 Gy [7]. The pain relief rate was 46.2% (6/13). LC was 3–14 months with a median of 7 months (95% CI: 3.5–10.5 months). The median survival was 10 months (95% CI: 3.9–16.1 months). Two (15.4%) patients experienced a grade 4 toxic event. Similarly, Wang et al. studied 20 patients with LRRC who were treated with CT-guided ^125^I seed implantation [24]. 14 of the 20 patients had history of adjuvant EBRT. The median matched peripheral dose was 120 Gy. The pain relief rate was 85% (17/20). Median survival time was 18.8 months (95% CI: 3.5–22.4 months). The local tumor control rate was 90%. Furthermore, one- and two-year local control rates were 65.0 and 15.0%, respectively. Two patients had mild hematochezia.

However, traditional RIS is implanted under direct visualization during the operation or under the guidance of imaging. The efficacy of the implantation significantly relies on the operators’ experience. Any unimplanted tumor area would be insufficiently covered by radiation dose owing to the sharp falloff of dose. The distribution of the dose could be inaccurate and the outcomes were unsatisfactory. Besides, the misplaced radioactive seeds may radiate OARs excessively and result in severe adverse effects. Considering the above-mentioned defects, we employed a 3D template for RIS implantation to avoid “hot” or “cold” spots. Postoperative dosimetric parameters can be highly consistent with the preoperative plan. The dose distribution can be highly conformal with the shape of the tumor to achieve persistent LC without damaging surrounding tissues. In the present study, the patients’ symptoms were remarkably relieved after the implantation (85.1% of patients experienced pain relief). Urinary symptoms, such as dysuria and frequent micturition were also relieved to some extent. Side effects greater than or equal to grade 3 occurred in 9.1% of patients, which were not only closely related to the dose of OARs, but also relevant to previous treatments. Although all the patients had previously received surgery or EBRT, they all exhibited a satisfactory tolerance. To our knowledge, there is no consensus about the relationship between dosimetric parameters and the prognosis of ^125^I implantation for LRRC. And the optimal dose of ^125^I implantation for tumors except for prostate cancer has still remained elusive. For the first time, we, in the present study, analyzed the optimal values of dosimetric parameters for the treatment of LRRC. The univariate regression analysis uncovered that D90, D100, and V100 were significant factors for LC. According to the ROC curve, the inflection points for D90, D100, and V100 were 130 Gy, 55 Gy, and 90%, respectively. Patients with LRRC who were treated with ^125^I implantation may have longer LC time when D90 > 130 Gy, D100 > 55 Gy, and V100 > 90%. Previous studies concentrating on prostate cancer reported that when V100 > 90%, LC time was markedly prolonged [27, 28], which was consistent with our results. Nevertheless, none of the dosimetric parameters had an effect on OS. This might be related to the following reasons. Firstly, the patients received different treatments after seed implantation. A number of patients were well tolerated and underwent targeted therapy or chemotherapy. However, some patients did not receive any treatment. Secondly, the majority of patients died of distant metastasis rather than local recurrence, indicating that systematic treatment after seed implantation should be warranted. Last but not least, the small sample size and retrospective nature of the study may also lead to the low impact of seeds implantation on OS.

## Conclusions

In summary, our results may provide a reliable reference for accurate treatment of LRRC patients based on traditional ^125^I implantation. Thanks to the development of technology, individualized 3D template was combined with CT-guided ^125^I implantation, which was proved to be a safer and more effective treatment for LRRC compared with other treatment methods. Patients’ refractory symptoms were remarkably relieved with mild side effects. Furthermore, a preliminary research on the optimal values of dosimetric parameters of ^125^I implantation for LRRC was undertaken. However, prospective studies with a larger sample size are required to further investigate the efficacy, adverse effects, and dosimetric factors related to RIS implantation with the assistance of 3D-PNCT.

## Data Availability

The datasets used and/or analyzed during the current study are available from the corresponding author on reasonable request.

## Abbreviations

LRRC: Locally recurrent rectal cancer
DFS: Disease free survival
EBRT: External beam radiotherapy
R0 resection: Radical resection
CT: Computed tomographic
RIS: Radioactive ^125^I seed
3D: Three-dimensional
3D-PNCT: 3D-printing non-co-planar template
B-TPS: Brachytherapy treatment planning system
GTV: Gross tumor volume
OARs: Organs at risk
D90: Dose delivered to 90% of GTV
D100: Dose delivered to 100% of GTV
V100: Percentage of GTV receiving 100% of the prescription dose
V150: Percentage of GTV receiving 150% of the prescription dose
V200: Percentage of GTV receiving 200% of the prescription dose
CI: The conformal index
EI: External index
HI: Homogeneity index
CR: Complete response
PR: Partial response
SD: Stable disease
PD: Progressive disease
LC: Local control
OS: Overall survival
ROC: Receiver operating characteristic
IORT: Intraoperative radiotherapy
MPD: Minimal peripheral dose
PET-CT: Positron emission tomography-computed tomography
